# Effect of chlorophyll biosynthesis-related genes on the leaf color in *Hosta* (*Hosta plantaginea Aschers*) and tobacco (*Nicotiana tabacum L.*)

**DOI:** 10.1186/s12870-020-02805-6

**Published:** 2021-01-15

**Authors:** Jingying Zhang, Changhai Sui, Huimin Liu, Jinjiao Chen, Zhilin Han, Qian Yan, Shuying Liu, Hongzhang Liu

**Affiliations:** 1 College of Life sciences, Jilin Agricultural University, 2888 Xincheng Street, Changchun City, 130000 People’s Republic of China; 2 Jilin Engineering Vocational College, Siping City, Jilin 136000 People’s Republic of China

**Keywords:** *HrHEMA*, *HrPOR*, *HrCAO*, Chlorophyll, Transgenosis, *Hosta*, Tobacco

## Abstract

**Background:**

‘Regal Splendour’ (Hosta variety) is famous for its multi-color leaves, which are useful resources for exploring chloroplast development and color changes. The expressions of chlorophyll biosynthesis-related genes (*HrHEMA, HrPOR* and *HrCAO*) in *Hosta* have been demonstrated to be associated with leaf color. Herein, we isolated, sequenced, and analyzed HrHEMA, HrPOR and HrCAO genes. Subcellular localization was also performed to determine the location of the corresponding enzymes. After plasmid construction, virus-induced gene silencing (VIGS) was carried out to reduce the expressions of those genes. In addition, *HrHEMA-, HrPOR-* and *HrCAO-*overexpressing tobacco plants were made to verify the genes function. Changes of transgenic tobacco were recorded under 2000 lx, 6000 lx and 10,000 lx light intensity. Additionally, the contents of enzyme 5-aminolevulinic acid (5-ALA), porphobilinogen (PBG), chlorophyll a and b (Chla and Chlb), carotenoid (Cxc), superoxide dismutase (SOD), peroxidase (POD), malondialdehyde (MDA), proline (Pro) and catalase (CAT) under different light intensities were evaluated.

**Results:**

The silencing of *HrHEMA, HrPOR* and *HrCAO* genes can induce leaf yellowing and chloroplast structure changes in *Hosta*. Specifically, leaves of *Hosta* with *HrCAO* silencing were the most affected, while those with *HrPOR* silencing were the least affected. Moreover, all three genes in tobacco were highly expressed, whereas no expression was detected in wild-type (WT). However, the sensitivities of the three genes to different light intensities were different. The highest expression level of *HrHEMA* and *HrPOR* was detected under 10,000 lx of illumination, while *HrCAO* showed the highest expression level under 6000 lx. Lastly, the 5-ALA, Chla, Cxc, SOD, POD, MDA, Pro and CAT contents in different transgenic tobaccos changed significantly under different light intensities.

**Conclusion:**

The overexpression of these three genes in tobacco enhanced photosynthesis by accumulating chlorophyll content, but the influential level varied under different light intensities. Furthermore, *HrHEMA-, HrPOR-* and *HrCAO-* overexpressing in tobacco can enhance the antioxidant capacity of plants to cope with stress under higher light intensity. However, under lower light intensity, the antioxidant capacity was declined in *HrHEMA-, HrPOR-* and *HrCAO-* overexpressing tobaccos.

**Supplementary Information:**

The online version contains supplementary material available at 10.1186/s12870-020-02805-6.

## Highlights

1. Silencing *HrHEMA*, *HrPOR* and *HrCAO* changed the cell permeability and resulted in accumulation of autophagic in vesicles.
2. Overexpressing of *HrHEMA*, *HrPOR* and *HrCAO* in tobacco enhanced photosynthesis by accumulating chlorophyll content, but the influential level deferred under different light intensity.
3. *HrCAO* overexpressing promoted flowering in tobacco plants.
4. Overexpressing of *HrHEMA*, *HrPOR* and *HrCAO* in tobacco can enhance the antioxidant capacity of plants to cope with stress under higher light intensity.

## Background

*Hosta (Hosta plantaginea* Aschers*)* is a perennial herbaceous and shade-loving plant of the family Liliaceae [1]. Its leaves vary in shape, size, color and texture. Furthermore, the cost of cultivation and maintenance of *Hosta* is relatively low, all of which will make it be widely used in urban greening and landscape construction [2]. Despite the ornamental value, *Hosta* plants also have important medicinal properties including heat-clearing and detoxifying functions. For instance, Liu noted that steroidal saponins extracted from the flower of *Hosta* exhibited anti-tumor effects [3]. Notably, the ‘*Regal Splendour*’, a *Hosta* variety, is famous for its multi-color leaves. The variegated leaves usually consist of green and white/yellow sectors, which are useful resources for studying chloroplast development and color changes [4]. Moreover, variegated leaf cells of variegated plants enumerated different characteristics with green eaves such as the chloroplast ultrastructure [5–7], construction and function of photosystem I (PSI) and photosystem II (PSII) [8, 9], and chlorophyll synthesis and degradation [5], which may mediate leaf color changing. Also, albinism plays a significant role in the changes in leaf color [10, 11]. Studies have reported that the expression alteration of each gene related to chloroplast may affect the biogenesis of chloroplasts. The resultant disruption in chlorophyll metabolism and chloroplast assembly can lead to abnormal leaf color [12, 13]. In chlorophyll biosynthesis, the *HEMA* gene encodes glutamyl-tRNA reductase (GluTR), which is one of the key regulators for encoding enzyme 5-aminolevulinic acid (5-ALA). The enzyme 5-ALA is synthesized from glutamate through three enzymatic reactions. Firstly, glutamate is converted to glutamyl adenylate and then transferred to specific tRNA to form glutamyl-tRNA. Thereafter, the GluTR catalyzes glutamyl-tRNA. to Glu 1-semialdehyde (GSA) in an NADPH-dependent reaction, and thus 5-ALA is formed by GSA aminotransferase [14]. Elsewhere, Kumar found that HEMA1 loss-of-function mutants presented patchy to completely yellow color and failed to thrive under normal growth conditions [15]. Another study by Schmied et al. elucidated that HEMA1 overexpression in etiolated and dark-grown plants can lead to protochlorophyllide accumulation [16]. Subsequently, the reduction of protochlorophyllide (Pchlide) to chlorophyllide, catalyzed by the enzyme protochlorophyllide oxidoreductase (POR), is the key light-driven reaction that lead to a profound transformation in plant development [17–20]. As the first step of light-dependent process in chloroplast biosynthesis, the expression of *POR* is related to the content of chlorophyll. In angiosperms, two POR isoforms (PORA and PORB) were first identified in *Hordeum vulgare* [21], *Arabidopsis (Arabidopsis thaliana)* [22], and some other plant species. Additionally, PORA transcripts up-regulated in young etiolated seedlings and down-regulated by light; whereas PORB transcripts were detected in dark-grown seedlings, as well as still remain detectable at later stages of development in the light [23]. The ternary complex formed by Chla, NADPH and POR is critical to the transformation from white body to chloroplast. Several studies has demonstrated that under the action of Chl synthase, Chla is eventually formed [24, 25]. In addition, Chlb is synthesized from Chla in a two-step oxygenation reaction catalayzed by Chla oxygenase (CAO) [26]. *HEMA, POR* and *CAO* all play a vital role in the process of chlorophyll synthesis and are closely related to leaf color. Their expression level may be involved in the regulation of plant leaf color. Herein, we sought to explore the functions of *HrHEMA, HrPOR* and *HrCAO* through gene silencing and overexpressing techniques in *Hosta* and tobacco plants. Lastly, the sensitivity of transgenic plants to light was studied under different light intensities. This study provides new evidences for the variegated leaves in *Hosta*.

## Results

### Identification of HrHEMA, HrPOR and HrCAO

**Fig. 1:**
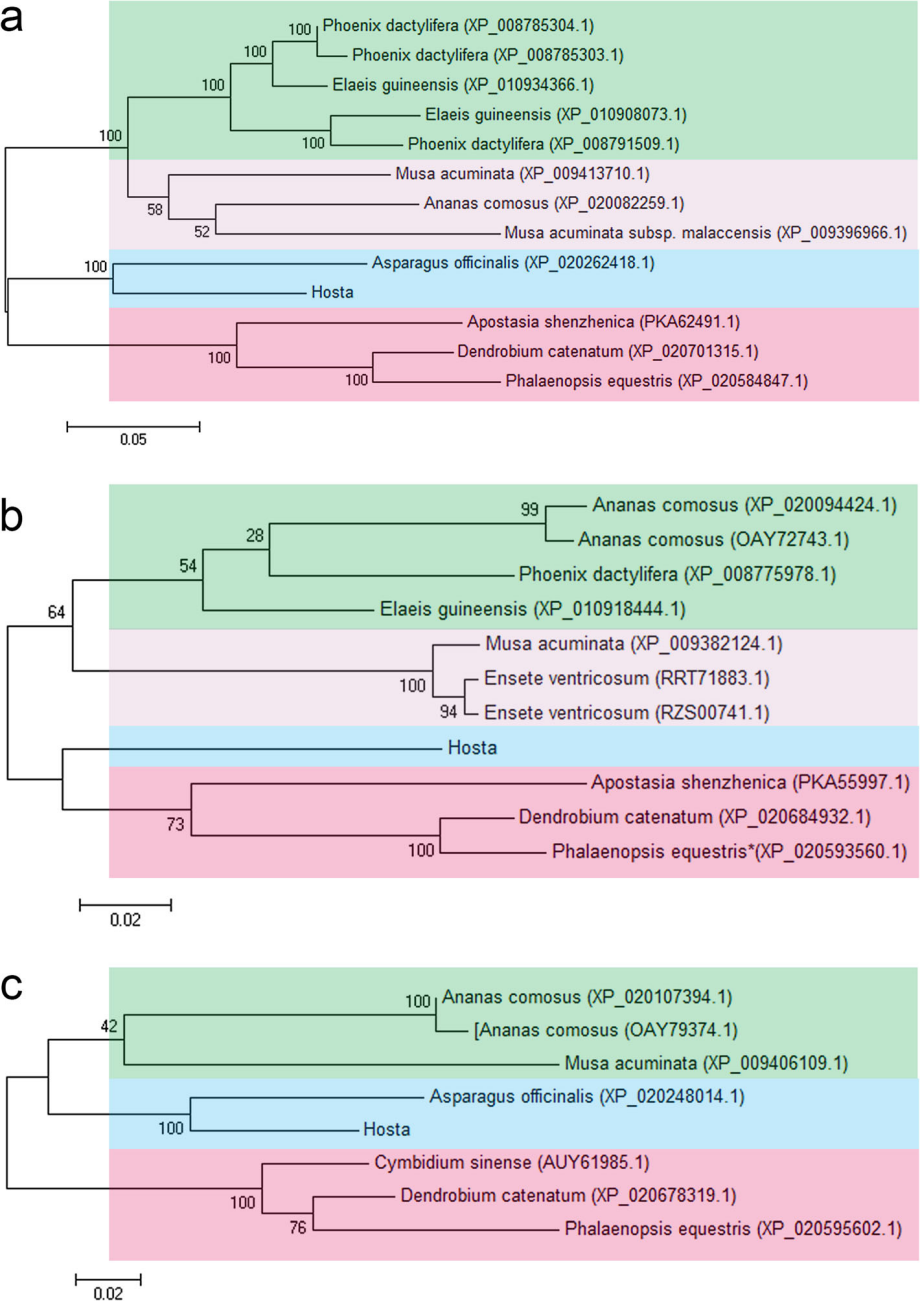
Evolutionary relationship results of *HrHEMA, HrPOR* and *HrCAO* from different species. **a**, **b**, and **c** note the phylogenetic trees of *HrHEMA, HrPOR* and *HrCAO* from different species, respectively. Different colors represent different clustering groups

The full-length of *HrHEMA, HrPOR* and *HrCAO* was 1608, 1185 and 1623 bp (**Table** **S1**), which encoded 536, 394 and 540 amino acids, respectively. Notably, sanger sequence results of the three genes are identical to those of the reference from NCBI **(Fig.** **S1****,** **S2****and** **S3**). Besides, the maximum likelihood (ML) trees were constructed based on the Sanger results and sequences obtained in NCBI using MEGA 7.0 (Fig. 1a-c). The phylogenetic trees revealed that all HrHEMA and HrCAO protein queries were clustered into four and three groups, respectively. *Hosta* was clustered together with *Asparagus officinalis* (Liliaceae), indicating that genes *HEMA* and *CAO* in *Hosta* and *A. officinalis* were more similar than those in other plants. For *HrPRO*, the protein queries were clustered into four groups, while no other plants were clustered with *Hosta*. Collectively, the relationship enumerated that the results of the phylogenetic tree were consistent with those obtained using the the Angiosperm Phylogeny Group (APG) IV system.

### Subcellular localization

**Fig. 2:**
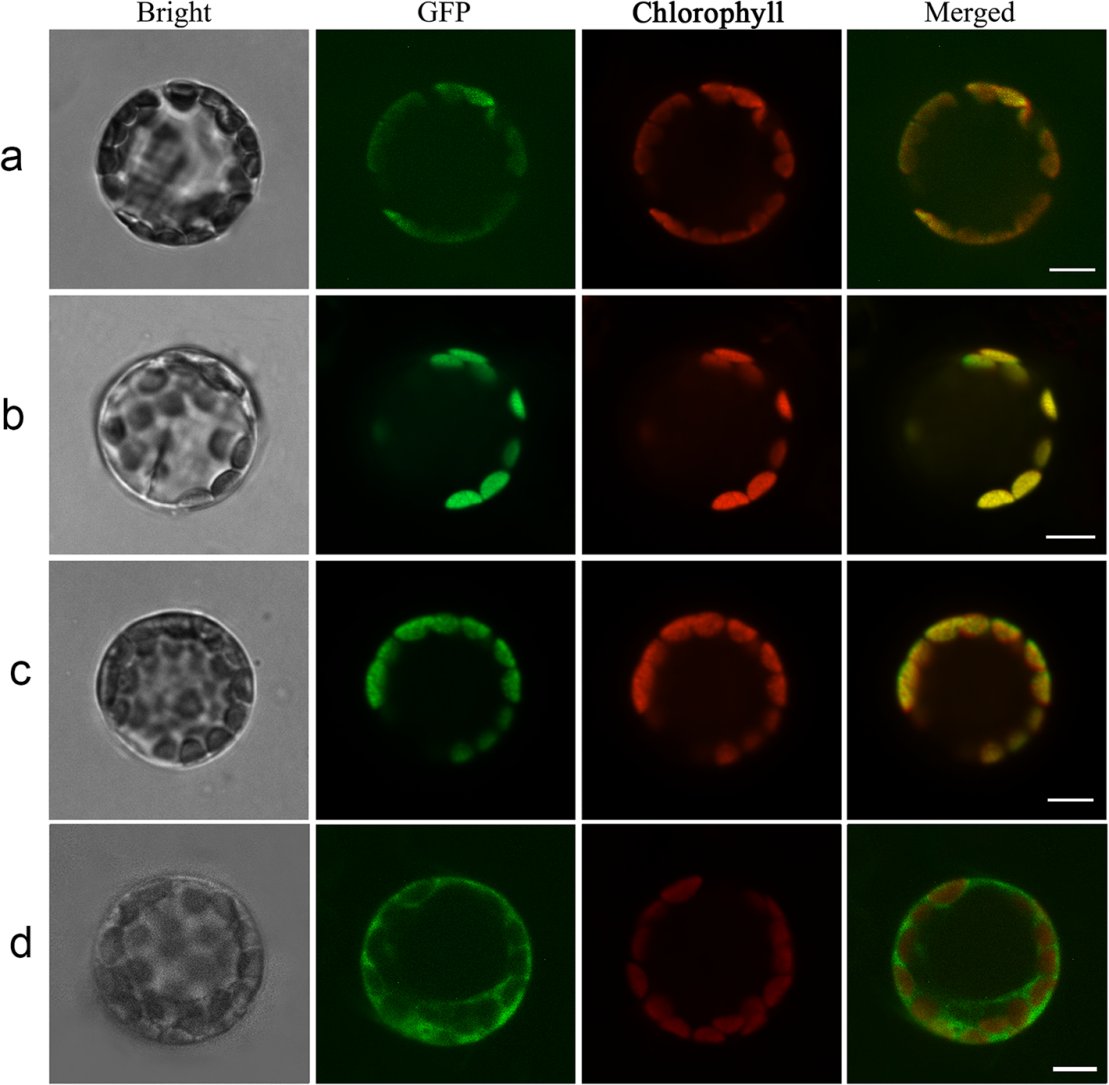
Subcellular localization of *HrHEMA, HrPOR* and *HrCAO* in *Arabidopsis thaliana* protoplasts. **a**, **b**, **c**, and **d** indicates *Arabidopsis thaliana* protoplast transformation with 16,318 h-HrHEMA-GFP, 16318 h-HrPOR-GFP, 16318 h-HrCAO-GFP and GFP empty vectors, respectively. Protoplasts in bright field (first column), green fluorescence (second column) and red autofluorescence (third column) were photographed. Merged images are shown in the fourth column. *Bright: bright field; GFP: green fluorescent protein; Chlorophyll: chlorophyll fluorescence

In order to identify the subcellular localization of the HrHEMA, HrPOR and HrCAO proteins, the full-length coding sequence (CDS) of the *HrHEMA*, *HrPOR* and *HrCAO* genes was cloned into an expression vector containing the GFP tag (16,318 h-GFP, 35S:GFP), separately. The 16,318 h-HrHEMA-GFP, 16318 h-HrPOR-GFP and 16,318 h-HrCAO-GFP fusion proteins were mainly distributed in chloroplast (higher brightness) (Fig. 2a-c). While GFP expressed from the empty expression vector (16,318 h-GFP, 35S:GFP), which served as a control, was mainly distributed in both chloroplast and nucleus (Fig. 2d). The distribution of the fusion proteins indicated that all the three proteins mainly located in the chloroplasts. This result was consistent with that predicted by bioinformatics.

### Effect of silenced HrHEMA, HrPOR and HrCAO on *Hosta plantaginea*

**Fig. 3:**
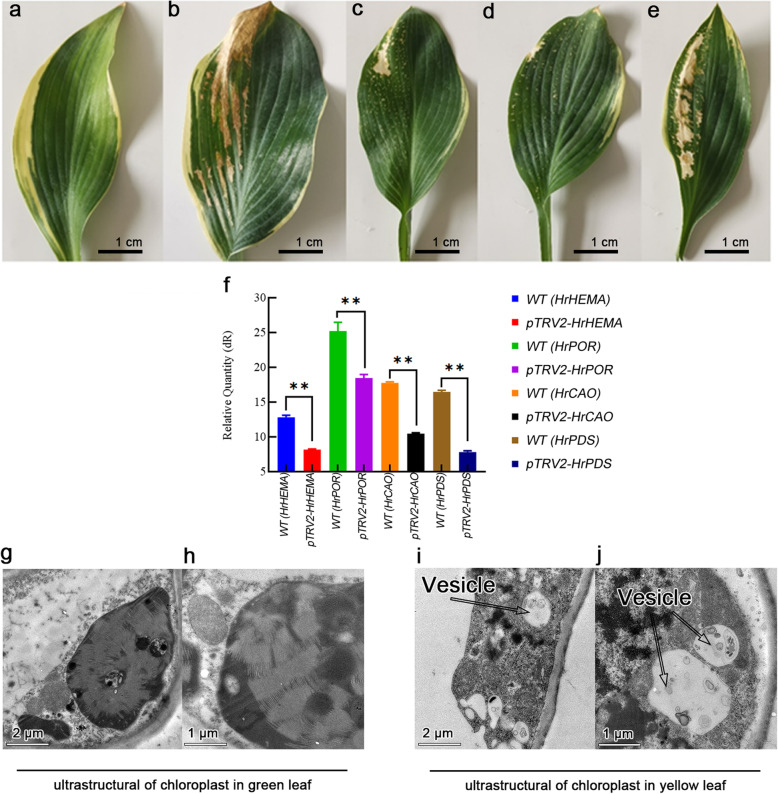
Effect of silencing *HrHEMA*, *HrPOR* and *HrCAO* on *Hosta plantaginea*. **a** to **e** note the changes of leaf color in control (negative control), *HrPDS* (positive control), *HrHEMA*, *HrPOR* and *HrCAO* science *Hosta* plant, respectively; **f** shows the gene expression of *HrHEMA*, *HrPOR, HrCAO* and *HrPDS*, ** means *p* < 0.05 compared with control (leaves without VIGS treatment); **g** and **h** show the ultrastructure of chloroplast in green leaf part. No vesicle (autophagosome) was found in green leaf part; **i** and **j** show the ultrastructure of chloroplast in yellow leaf part. Some vesicles (autophagosome) were found in the in yellow leaf part

Silencing of *HrHEMA, HrPOR and HrCAO* genes in *Hosta* resulted in leaf albino sectors 40 days after VIGS inoculation (Fig. 3a-e). The *HrPDS* was used as a positive control, whereas untransformed leaves were used as a negative control. Generally, the findings showed that different silenced genes caused various degrees of albinism in plants. Specifically, similar to the positive control, the *HrCAO*-silenced plant showed the highest rates of albinism. While the *HrPOR* silencing plant depicted the lowest one. Additionally, the expression levels of *HrHEMA, HrPOR and HrCAO* were significantly decreased after VIGS silencing treatment compared to the control. In particular, *HrHEMA* was decreased by 36.2%, *HrPOR* by 26.8%, *HrCAO* by 41% and *HrPDS* by 52.5% (Fig. 3f). The ultrastructural observations results demonstrated that the cell permeability was changed, and many vesicles (autophagosome) appeared in the yellow part of leaves (Fig. 3g-j). The chloroplasts transformed from normal ellipses to roundness, thylakoids changed from flattened to swollen shape, and the inner membrane system was incomplete. Finally, mitochondria cristae were aggregated and well-developed after chlorophyll biosynthetic gene silencing.

### Expression patterns of HrHEMA, HrPOR and HrCAO in tobacco leaves under different light intensities

**Fig. 4:**
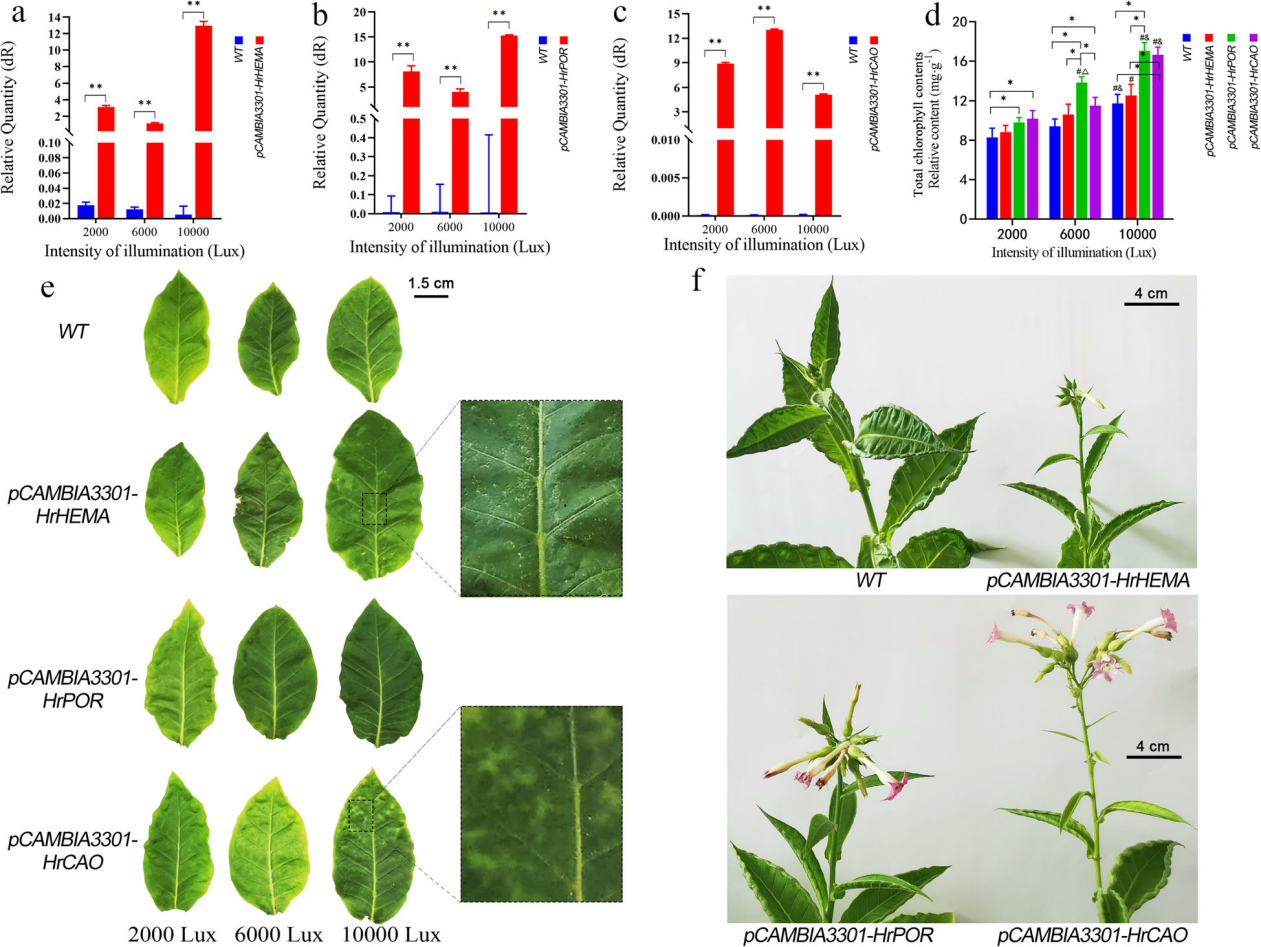
Effect of overexpression *HrHEMA*, *HrPOR* and *HrCAO* on Tobacco. **a** to **c** show changes in expression of *HrHEMA*, *HrPOR* and *HrCAO* in transgenic tobacco leaves. ** means *p* < 0.05 compared with control; **d** Total chlorophyll contents of all the transgenic tobacco leaves. * means *p* < 0.05; # means *p* < 0.05 compared with 2000 lx; # means *p* < 0.05 compared with 600 lx; **e** shows the leave color changes of *HrHEMA*, *HrPOR* and *HrCAO* transgenic tobacco leaves under 2000 lx, 6000 lx and 1000 lx illumination. Images were photographed 40 days after different light intensities; **f** exhibits the plant status after 70 days growth under 10,000 lx light intensity

In overexpressing tobacco plants, the expression level of *HrHEMA, HrPOR* and *HrCAO* were significantly higher than those of WT plants. Different genes overexpressing tobacco plants exhibited different sensitivities to light. The highest expression level of *HrHEMA* and *HrPOR* was detected under 10,000 lx of illumination, while *HrCAO* showed the highest expression level under 6000 lx (Fig. 4a-c). Furthermore, it was noted that the total chlorophyll content in *HrPOR-* and *HrCAO-*overexpressing in tobacco plants reached a peak under 6000 lx and 10,000 lx, respectively (Fig. 4d). After treating WT and transgenic tobacco plants with different light intensities (2000 lx, 6000 lx and 10,000 lx) for 40 days, it was noted that all tobacco leaves exhibited considerable changes (Fig. 4e). With the increase of light intensity, the leaf color gradually changed from light green to dark green. At 10000 lx, the leaf color was relatively close to that under sunlight. Besides, palisade and spongy tissues appeared in *HrHEMA* transgenic leaves, where light spots appeared on the surface of *HrCAO* transgenic tobacco leaves. After 70 days, substantial differences in plant growth were recorded under different light intensities, and specifically, the fastest growth rate was under 10,000 lx, followed by 6000 lx and 2000 lx. Moreover, the budding time of transgenic tobacco under 10,000 lx illumination was earlier compared to that of the control. Interestingly, the order of flowering time was as follows, the *HrCAO* transgenic tobacco was the earliest, then the *HrPOR* and *HrHEMA* transgenic tobacco, and finally the *WT* tobacco (Fig. 4f).

### Changes of chlorophyll biosynthetic precursors in HrHEMA-, HrPOR- and HrCAO-overexpressing tobacco seedlings

**Fig. 5:**
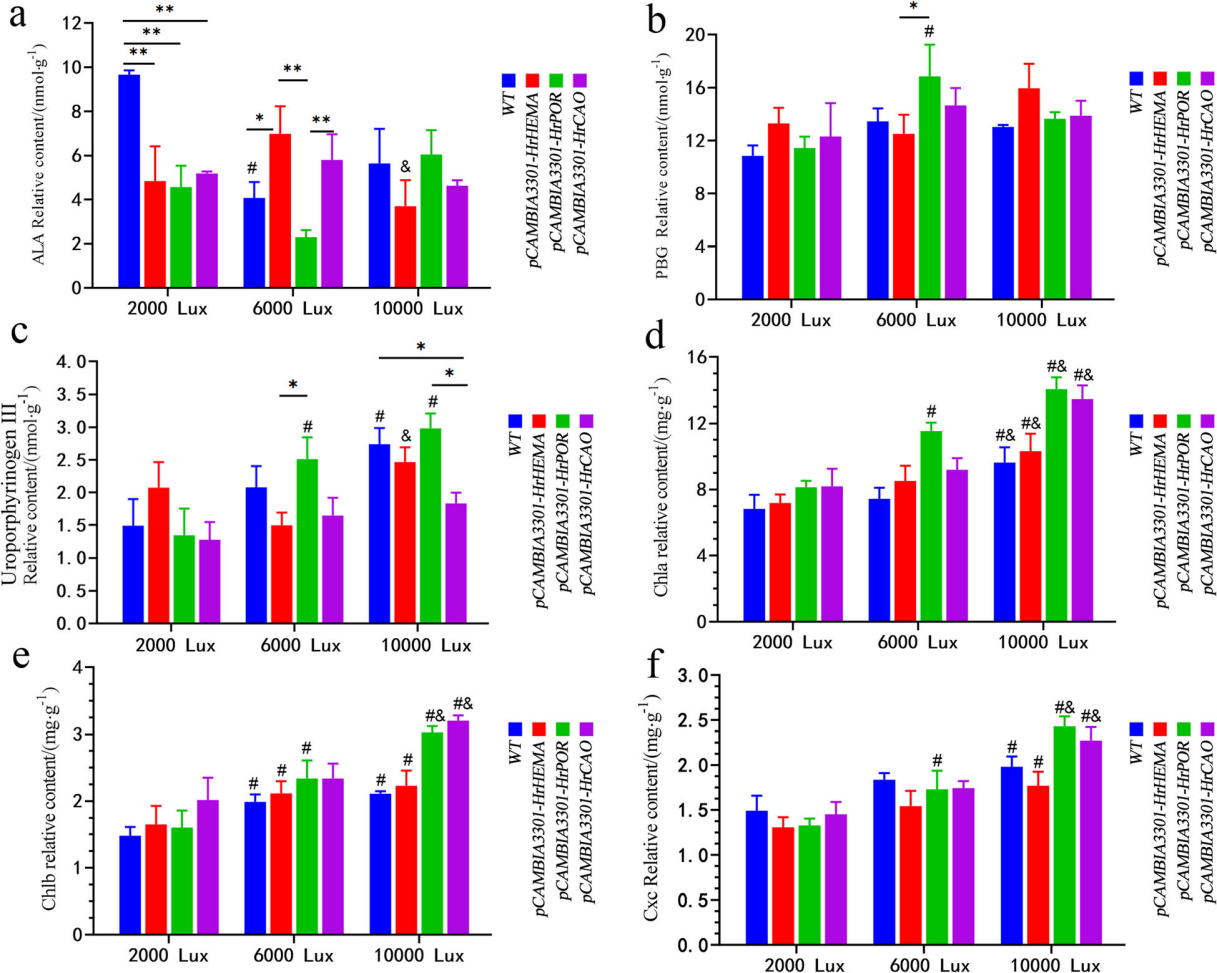
Content of 5-aminolevulinic acid (5-ALA), porphobilinogen (PBG), uroporphyrinogen III, Chlorophyll a and b (Chla and Chlb) under different light intensities in *HrHEMA*, *HrPOR* and *HrCAO* transgenic tobacco leaves. **a** to **f** show the contents of 5-ALA, PBG, uroporphyrinogen III, Chla and Chlb, respectively. * represents *p* < 0.05, ** represents *p* < 0.01; # represents *p* < 0.05, comparison between 6000 (or 10,000) and 2000 lx of the same gene; & represents *p* < 0.05, comparison between 10,000 and 6000 lx of the same gene

Here, the enzyme 5-ALA, as the first universal precursor in the synthesis of chlorophyll, heme and other tetrapyrroles, revealed a relatively higher content in control than in the transgenic plants under 2000 lx. Under 6000 lx illumination, *HrHEMA- and HrCAO-* overexpressing plants possessed a higher content of 5-ALA than those of *HrPOR*. On the contrary, *HrPOR-*overexpressing plants showed a higher 5-ALA than the *HrHEMA and HrCAO* transgenic plants under 10,000 lx (Fig. 5a). For porphobilinogen (PBG), no significant difference was noted under 2000 lx illumination. However, PBG in *HrPOR-*overexpressing tobacco leaves elucidated the highest relative content under 6000 lx, while that in *HrHEMA* transgenic tobacco leaves had the highest one under 10,000 lx (Fig. 5b). With the increase of light intensity, the uroporphyrinogen III content of WT, *HrPOR-* and *HrCAO-* overexpressing tobacco plants depicted an increasing trend. Additionally, the uroporphyrinogen III content of all the plants reached a peak under the light condition of 10,000 lx. On the other hand, the uroporphyrinogen III in *HrHEMA* overexpressing tobacco was first decreased and then increased. While in *HrPOR* transgenic tobacco plants, uroporphyrinogen III was increased with the light intensity (Fig. 5c).

### Effect of overexpression of chlorophyll biosynthetic genes on Chla, Chlb and cxc

The contents of Chla and Chlb increased gradually with light intensity and were higher in transgenic tobacco than those of WT under the same light condition. Furthermore, the Chla and Chlb contents of *HrPOR-* and *HrCAO-* overexpressing plants were increased considerably, especially under 10,000 lx illumination (Fig. 5d and e). The content of Cxc increased with the light intensity, but no significant difference was found among different groups under 2000 and 6000 lx illumination (Fig. 5f). However, under 10,000 lx illumination, the contents of Cxc in *HrPOR* and *HrCAO* transgenic tobacco plants were statistically higher than those in WT.

### Effect of overexpression of chlorophyll biosynthetic genes on stress adjustment compounds

The contents of SOD, POD, MDA, Pro and CAT under different light conditions are summarized in Table 1. Under low light intensity, the SOD content in WT was higher than other groups. While under high light intensity, the SOD in *HrCAO* and *HrPOR* transgenic plants were relatively higher than others. POD illustrated a negative correlation with light intensity, while different gene overexpression had different effects. Especially in *HrPOR* transgenic plants, the POD content was significantly lower compared to other groups. For MDA, the contents in *HrCAO* and *HrPOR* transgenic plants and WT showed a negative correlation with light intensity, whereas those in *HrHEMA*-overexpressing plants displayed a positive correlation. Moreover, in general, the Pro contents in all the plants was negatively correlated with light intensity. Additionally, all the tobacco plants had significantly higher Pro contents under 2000 and 6000 lx illumination than that under 10,000 lx. Finally, the CAT contents in all the transgenic tobacco plants were increased with light intensity, while the WT tobacco showed an opposite trend.

**Table 1:** Contents of SOD, POD, MDA, Pro and CAT

	Illumination	Control	HrHEMA	HrPOR	HrCAO
SOD (nmol·g^−1^)	2000 Lux	457.95	410.17	394.26	331.70
	6000 Lux	322.87	317.56	372.31	343.21
	10,000 Lux	277.04	277.40	421.99	401.92
POD (nmol·g^−1^)	2000 Lux	4582.12	3870.28	1533.59	5521.09
	6000 Lux	3123.75	2880.60	832.71	2722.97
	10,000 Lux	2360.76	1241.45	330.64	588.15
MDA (nmol·g^−1^)	2000 Lux	32.31	23.67	38.67	23.37
	6000 Lux	23.50	26.30	28.66	42.95
	10,000 Lux	20.31	31.88	23.37	31.18
Pro (mg·g^−1^)	2000 Lux	22.37	31.38	30.98	27.95
	6000 Lux	19.18	21.99	21.62	19.24
	10,000 Lux	12.42	12.30	11.49	10.59
CAT (nmol·g^−1^)	2000 Lux	255.42	40.90	102.98	62.10
	6000 Lux	152.67	115.80	241.02	136.24
	10,000 Lux	27.63	267.23	325.05	275.84

## Discussion

The color change of plant leaves is a complex and sensitive process, controlled by a variety of genes, transcription factors and metabolic pathways. The color of plant leaves is attributed to the chlorophyll content, which is regulated by multiple genes, including *HEMA, GSA, HEME, POR, CAP*, *CHLG* and *CHLM* [27]. Previous studies have demonstrated a relationship between the expression levels of *HrHEMA, HrPOR* and *HrCAO* and leaf color in plants [28–30]. In this respect, we focused on the functions of *HrHEMA, HrPOR* and *HrCAO* and their relationship with light in *Hosta*.

Furthermore, *HrHEMA, HrPOR* and *HrCAO* genes, encoding the key enzyme for 5-ALA for chlorophyll synthesis, were isolated and characterized. As anticipated, the sanger sequence results of the three genes matched the reference gene sequence perfectly, indicating that the isolated genes were correct. Our phylogenetic tree outcomes revealed that *Hosta* had a closer relationship with *Asparagus officinalis* than other species, which is consistent with previous studies [31, 32]. Also, the phylogenetic trees agree well with the classification of the APG IV system [33]. The subcellular localization results revealed that all these three proteins were located in chloroplasts, which corroborates consistent with the results of previous studies [34–36].

After gene silencing, the expression levels of *HrHEMA, HrPOR* and *HrCAO* were decreased significantly. Besides, the color of *Hosta* leaves changed significantly, which indicated that the expressions of *HrHEMA, HrPOR* and *HrCAO* in *Hosta* can directly affect the change of leaf color. However, silencing of different genes had different effects on the degree of leaf color changes. For example, the silencing of *HrCAO* mediated the formation of a large number of white spots in leaf. Chlb is one of the major light-harvesting pigments produced by land plants, green algae and several cyanobacterial species, which is synthesized from Chla by *CAO* [37]. The chlorophyll cycle plays a crucial role in the processes of greening, acclimation to light intensity and senescence [38]. Studies have reported that the reduced expression level of *HrCAO* led to a decrease of Chlb, which might seriously interfere with the chlorophyll cycle and thus result in the leaf discoloration. On the other hand, the leaf color changed from light green to dark green in the *HrCAO*-overexpressing tobacco plants, and mottled leaves appeared at 10000 lx. In another study, the *CAO*-overexpressing tobacco plants have been reported to photosynthesize for a longer duration and retain larger biomass [39]. Overexpressing of a modified *Arabidopsis* CAO gene results in excessive accumulation of Chlb [40, 41]. It is likely that the overexpression of *HrCAO* in tobacco mediates the accumulation of Chlb and retains larger biomass. Thus, the color gradually darkens. The rapid accumulation of Chla and Chlb and total chlorophyll content at 10000 lx also supported the fact that the accumulation of chlorophyll deepens the leaves color. The effect of *HrPOR* gene silencing on leaf color was minimal of the three genes, and also the declining extent of expression level was the lowest. Of note, *HrPOR* silencing can cause white spots on the surface of leaves. On the contrary, its overexpression made the leaf color darker with the increase of light intensity.

The POR is a key regulatory enzyme in the chlorophyll synthesis pathway of all oxygen-producing photosynthetic organisms, participating in the enzymatic reduction of protochlorophyllide to chlorophyll by light. This is the first step in light-dependent chloroplast biosynthesis [42–44]. Multiple studies have noted that the light saturation point of tobacco seedlings is about 10,000 lx (~ 178.6 μmol·m^− 2^·s^− 1^), while that for tobacco at the adult plant stage is about 30,000 lx (~ 535.7 μmol·m^− 2^·s^− 1^) [45, 46]. According to our results, the PBG content under 6000 lx showed the maximum value, which indicated that the tobacco with *HrPOR* overexpression has faster photosynthetic rates while the peak light demand is lower. Elsewhere, in plant cells, PBG was converted to uroporphyrinogen III by uroporphyrinogen synthase (UROS) [47]. Here, the relative content of uroporphyrinogen III was increased rapidly under 6000 lx light, which was mediated by the increase of PBG. This further supported that HrPOR-overexpressing tobacco has a higher photosynthetic rate.

**Fig. 6:**
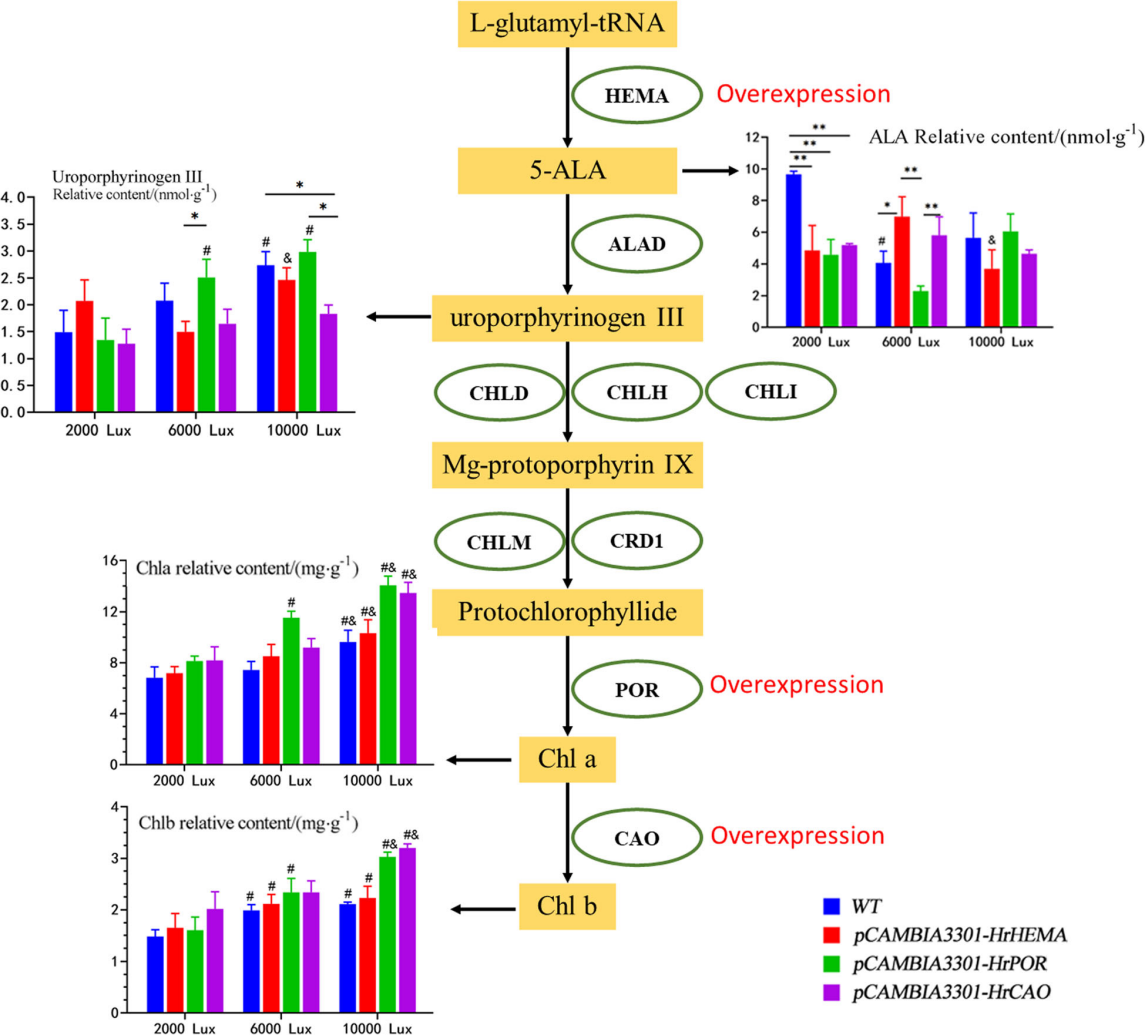
Summary of results. Ovals represent gene abbreviations. Rectangles represent key compounds. The histogram shows the relative content of key factors. Wild-type (*n* = 3) is shown in blue, *HrHEMA* overexpression (*n* = 3) is shown in red, *HrPOR* overexpression (*n* = 3) is shown in green, and *HrCAO* overexpression (*n* = 3) is shown in purple. * represents *p* < 0.05, ** represents *p* < 0.01; # represents *p* < 0.05, compared with 2000 lx; & represents *p* < 0.05, compared with 6000 lx of the same gene. ALA, aminolevulinic acid; Chla, chlorophyll a; Chlb, chlorophyll b

The contents of Chla and total chlorophyll in *HrPOR* transgenic tobacco were increased significantly under 6000 lx. Conversely, the contents of Chla, Chlb and total chlorophyll were increased significantly under 10,000 lx, which may be the main reason for the darkened green color. These findings are similar to the results of a previous study, where *AtPORC*-overexpression leads to a greener leaf phenotype in *Arabidopsis* [48]. Recently, several studies have reported that *POR* expression is induced by light, but shows different expression patterns in response to light [48–50]. In this study, *HrPOR* exhibited higher expression under 6000 lx, but the accumulation of chlorophyll reached its peak at 10000 lx, which indicated that moderate light intensity may be suitable for chlorophyll accumulation, and while excessive light intensity may induce opposite effects. The ALA is the precursor of Pchlide, which acts as a herbicide in the presence of light [51, 52]. A study found that *AhHEMA1-*overexpressing tobacco can effectively yield in a prominent increase in ALA biosynthesis, with higher chlorophyll contents [53]. Herein, our results revealed that *HrHEMA-*overexpressing tobacco had lower 5-ALA contents under 2000 and 10,000 lx than WT, but showed higher content of 5-ALA under 6000 lx. This signifies that the content of 5-ALA in WT maintained a high level under low light conditions, but after *HrHEMA* overexpression, the 5-ALA content was more sensitive to light and rapidly decomposed, even under low light conditions. In addition, the palisade and spongy tissues appeared in *HrHEMA* transgenic tobacco leaves, which was in line with the results reported by Schmied et al. [16], who recorded chlorosis and variegation in seedling growth under 160 μE long sunshine conditions. The changes in chloroplast ultrastructure uncovered that the down-regulated *HrHEMA, HrPOR* and *HrCAO* significantly affected the chloroplast structure, which may be a major reason for the changes of the leaf color. The contents of Chla, Chlb and chlorophyll in transgenic plants were increased compared with the WT tobacco, which may enhance photosynthesis, improve light energy utilization rate, and promote the growth of tobacco plants to a certain extent. Furthermore, the transfer of *HrHEMA, HrPOR* and *HrCAO* can shorten the growth cycle [54], particularly for *HrCAO* transgenic tobacco. Our results correspond with previous findings [55]. Here, we summarized our results in Fig. 6.

Reactive oxygen species (ROS) play an integral role as signaling molecules in the regulation of numerous biological processes such as growth, development and responses to biotic and/or abiotic stimuli in plants [56, 57]. The balance of ROS in plant cells is important for plant development. The antioxidant defense system in the plant cell includes all enzymes such as SOD, POD and CAT [58].

The content of MDA, produced during peroxidation of membrane lipids, is often used as an indicator of oxidative damages [59]. Whereas that of Pro in a plant is a common response indicator to stress [60, 61]. The relative contents of SOD, POD and CAT elucidated that the expressions of *HrHEMA, HrPOR* and *HrCAO* can greatly affect their relative contents. However, here we noted that transgenic tobacco plants can enhance the activity SOD and CAT under strong illumination conditions, which can eliminate ROS produced in the intense photosynthesis process. Besides, transgenic tobacco plants depicted stronger photosynthesis ability, which may lead to an increase of MDA and Pro contents. Those findings were mainly regulated by heme, which might be used as a prosthetic group to participate in oxidative homeostasis [62]. Overall, *HrHEMA-, HrPOR-* and *HrCAO-*overexpressing tobacco can enhance the antioxidant capacity of plants to cope with stress under higher light intensity. Nevertheless, under lower light intensity, the antioxidant capacity of *HrHEMA-, HrPOR-* and *HrCAO-* overexpressing in tobacco declined.

## Conclusion

In summary, *HrHEMA, HrPOR* and *HrCAO* silencing can cause leaf yellowing and chloroplast structure changes in *Hosta*. On note, leaves of *Hosta* with *HrCAO* silencing were the most affected, while the *HrPOR* silencing plant was the least affected. The overexpression of these three genes in tobacco enhanced photosynthesis by accumulating chlorophyll content, but the influential level varied under different light intensities. Furthermore, *HrHEMA-, HrPOR-* and *HrCAO-* overexpressing in tobacco can enhance the antioxidant capacity of plants to cope with stress under higher light intensity. However, under lower light intensity, the antioxidant capacity deteriorated in the *HrHEMA-, HrPOR-* and *HrCAO-*overexpressing tobaccos.

## Methods

### Plant materials, growth and treatments

*Hosta* (Lamarck cv. Regal Splendor), *Arabidopsis* (L. Heynh cv. Columbia) and tobacco (*Nicotiana tabacum* cv. NC89) plants were used in this study. *Hosta* were planted in the nurseries of Conservation and Exploitation of Wild Resources of Changbai Mountain, Jilin Agricultural University. Whilst *Arabidopsis* and tobacco were planted in an artificial climate chamber with illuminated conditions [4000 lx for *Arabidopsis*, 6000 lx for tobacco (tobaccos for illumination experiment were not included), 14 h/day] and mid-range humidity (40–60%). Fresh plant leaves of *Hosta* were sampled and stored at − 80 °C every 15 days. While those of *Arabidopsis* were used for protoplast preparation. For tobacco, seeds were disinfected with ethanol and sodium hypochlorite, and then sown on Murashige and Skoog (MS) media. After germination, seeds were transplanted in sterilized soil and grown in the artificial climate chamber for two weeks.

### Isolation of HrHEMA, HrPOR and HrCAO and sequence analysis

Total RNA in *Hosta* leaves was extracted using a mini BEST Universal RNA Extraction Kit (TAKARA, Shiga, Japan). Then, a PrimeScript™ RT reagent Kit 3.0 (TAKARA, Shiga, Japan) was used for reverse-transcription PCR (RT-PCR). Afterward, gDNA Eraser was used to remove DNA from total RNA (1 μg) with at 42 °C for 2 min, and then the transcription reaction mix was added and incubated at 37 °C for 15 min and terminated at 85 °C for 5 s. The PCR conditions used for *HrHEMA, HrPOR and HrCAO* amplification with the paired primers (**Table** **S2**) were 94 °C for 3 min; 30 cycles at 98 °C for 10 s, 62 °C for 5 s, 72 °C for 20 s; 72 °C for 10 min and held at 4 °C. The 5′- and 3′-fragments of these three genes were obtained from cDNA using the RACE Kit (TAKARA, Shiga, Japan). The obtained PCR fragments were subsequently cloned into the pEASY-Blunt Simple Cloning Vector (TransGen Biotech, Beijing, China). The recombinant plasmids were transformed into *E. coli Trans1*-T1, and white colonies were selected on LB plates containing 50.0 mg/mL Amp, 500 mg/mL IPTG and 20 mg/mL X-gal. To characterize the isolated genes, the sanger sequence was performed with the M13 primer (F: GAACACGGGGGACTCTTGAC; R: AATGTTTGAACGATCGGGGAAA) using an ABI 3730 xl sequencer (ABI, CA, USA).

### Construction of phylogenetic trees

The homologous sequences of *HrHEMA, HrPOR* and *HrCAO* were retrieved using Blast. The phylogenetic analysis of the ORF (Open Reading Frame) region for *HrHEMA, HrPOR* and *HrCAO* was executed, respectively, by comparing sequences and analyzing homology with homologous sequences. Briefly, the sequence multiple alignments were performed by the use of Clustal W with default parameters. Then the phylogenetic trees were constructed with the Maximum Likelihood (ML) algorithm using MEGA 6.0 (www.megasoftware.net/). Finally, the ML tree reliability was estimated using bootstrap analysis with 1000 replicates.

### Subcellular localization

We constructed a vector expressing a GFP-tagged fusion protein for subcellular localization analysis of the HrHEMA, HrPOR and HrCAO protein. The cDNA containing the coding sequence of *HrHEMA, HrPOR* and *HrCAO* were cloned into the 16,318 h GFP vector with a CaMV 35S promoter: green fluorescent protein (35S:GFP) cassette to construct fusion proteins following the previous report [63]. The recombinant vectors (*p35S:16318 h-HrHEMA-GFP*, *p35S:16318 h-HrPOR-GFP and p35S:16318 h-HrCAO-GFP*) were confirmed by sanger sequencing. The paired specific primers are listed in **Table** **S2**. The recombinant vectors were transformed into protoplasts using the polyethylene glycol (PEG)-mediated transformation method as described previously [64]. Fluorescence was examined under a Olympus FluoView FV1000 confocal laser scanning microscope (Olympus, Japan) 16 h after transformation.

### Plasmid construction and gene silencing in Hosta leaves

For the gene silencing experiment, *Hosta* was used as the plant material. The full-length cDNA of *HrHEMA, HrPOR* and *HrCAO* were inserted into VIGS vector pTRV2. Afterward, three VIGS expression vectors (*pTRV2-HrHEMA, pTRV2-HrPOR, and pTRV2-HrCAO*), and positive control, *pTRV2-PDS* (Phytoene Desaturase), were inoculated into the *Agrobacterium tumefaciens*. The *A. tumefaciens* with the recombined vectors were injected into the leaf and stalk with Agro liquid culture using a 1-ml syringe. After injection, the seedlings were cultured in an incubator at 16 °C with a relative humidity of 60%. After 24 h, the seedlings were cultured at 22/18 °C and 16 h day/8 h night. The qRT-PCR was performed 14 days later to measure gene expression in order to verify the effect of gene silencing. Lastly, after 40 days, the plant leaves were observed and recorded for bleaching.

### Observation of leaf ultrastructure

To observe the changes of the chloroplast in the different color parts of the *Hosta* leaf, the ultrastructure of a leaf was observed by transmission electron microscope (TEM, Hitachi, Tokyo, Japan). The center of the part to be detected was cut into small pieces (about 1 mm^3^) to ensure uniformity of samples. Then leaf tissues were fixed in 5% glutaraldehyde in 0.05 M phosphate buffer (pH 7.2), post-fixed in 2% osmium tetroxide in the same buffer, dehydrated with a graded acetone series and propylene oxide, embedded in resin (analytically pure), and polymerized at 65 °C for 24 h. Samples were then observed by TEM at 100 kV.

### Plasmid construction and agrobacterium-mediated transformation of tobacco plants

The full-length cDNAs of *HrHEMA, HrPOR,* and *HrCAO* were cloned into the expression vector pCAMBIA3301 under the control of CaMV 35S promoter (http://www.cambia.org). The gusA acted as the reporter gene, which is only expressed in eukaryotic cells. The vectors of *35S:pCAMBIA3301-HrHEMA, 35S:pCAMBIA3301-HrPOR, 35S:pCAMBIA3301-HrCAO*, and a control *pCAMBIA3301* were introduced into the *A. tumefaciens* strains using the freeze-thaw method. Leaf disk transformation was done based on the previous report [65]. After 2–3 weeks, the regenerated tobacco tissues were transferred to the medium for inducing roots. The qRT-PCR was employed for screening positive transgenic plants.

### Changes of transgenic tobacco under different light conditions

Wild-type tobacco (WT, control) and transgenic tobacco plants at the seedling stage were treated with different light intensities including 2000 lx, 6000 lx and 10,000 lx. The changes in leaves were observed after 40 days while the plant growth was monitored after 70 days. In addition, the contents of 5-ALA and porphobilinogen (PBG) and relative contents of uroporphyrinogen III, chlorophyll a and b (Chla and Chlb), carotenoid (Cxc), superoxide dismutase (SOD), peroxidase (POD), malondialdehyde (MDA), proline (Pro) and catalase (CAT) under different light intensities were assessed as described elsewhere [66, 67].

### Quantitative real-time polymerase chain reaction (qRT-PCR) analysis

Quantitative RT-PCR was implemented on an ABI 7500 Real-time PCR System using SYBR Real Master Mix (TAKARA, Shiga, Japan) with the following PCR thermal cycle conditions: denaturation at 95 °C for 30 s, 40 cycles at 95 °C for 5 s, 58 °C for 10 s, and 68 °C for 10 s. Additionally, Hv-Actin and Ntβ-Actin were used as the reference genes in *Hosta* and tobacco, respectively (primers used are outlined in **Table** **S2**). Template-free, negative and single primer controls were established before the examination. The results are presented in three biological replicates (each with three technical replicates) for each sample, and the 2^-ΔΔ CT^ method was used for statistical analysis.

### Statistical analysis

Statistical data analysis was executed following previous studies [68, 69]. In particular, GraphPad Prism 8 software was used for data statistical analysis. Data were analyzed using one-way ANOVA followed by multiple comparisons with the Tukey test. A value of *p* < 0.05 was considered significant, while *p* < 0.01 was considered highly significant.

## Supplementary Information

**Additional file 1: Table S1.** Basic information of *HrHEMA*, *HrPOR* and *HrCAO.*

**Additional file 2: Table S2.** Primer sequences of gene cloning.

**Additional file 3: Fig. S1.** Alignment and evolutionary relationship results of HrHEMA from different species.

**Additional file 4: Fig. S2.** Alignment and evolutionary relationship finding of HrPOR from different species.

**Additional file 5: Fig. S3.** Alignment and evolutionary relationship results of HrCAO from different species.

## Data Availability

We uploaded the data to GenBank with the direct link and login number: https://www.ncbi.nlm.nih.gov/nuccore/MW217998, HrCAO.sqn HrHEMA MW217998; https://www.ncbi.nlm.nih.gov/nuccore/MW217999, HrCAO.sqn HrPOR MW217999; https://www.ncbi.nlm.nih.gov/nuccore/MW218000, HrCAO.sqn HrCAO MW218000. The data deposited in the GenBank is publicly available.

## Abbreviations

PSI: photosystem I
PSII: photosystem II
GluTR: glutamyl-tRNA reductase
5-ALA: enzyme 5-aminolevulinic acid
GSA: Glu 1-semialdehyde
POR: protochlorophyllide oxidoreductase
CAO: Chla oxygenase
APG: Angiosperm Phylogeny Group
PBG: porphobilinogen
ROS: Reactive oxygen species
MS: Murashige and Skoog medium
RT-PCR: reverse-transcription PCR
PDS: Phytoene Desaturase
Chla and Chlb: Chlorophyll a and b
Cxc: Carotenoid
SOD: Dismutase
POD: Peroxidase
MDA: Malondialdehyde
Pro: Proline
CAT: Catalase
qRT-PCR: Quantitative real-time polymerase chain reaction
