# Supplementary figures and images for: Effect of chlorophyll biosynthesis-related genes on the leaf color in Hosta (Hosta plantaginea Aschers) and tobacco (Nicotiana tabacum L.)
Source: BMC Plant Biol. 2021 Jan 15;21:45. doi: 10.1186/s12870-020-02805-6 (PMC7811250; doi:10.1186/s12870-020-02805-6)

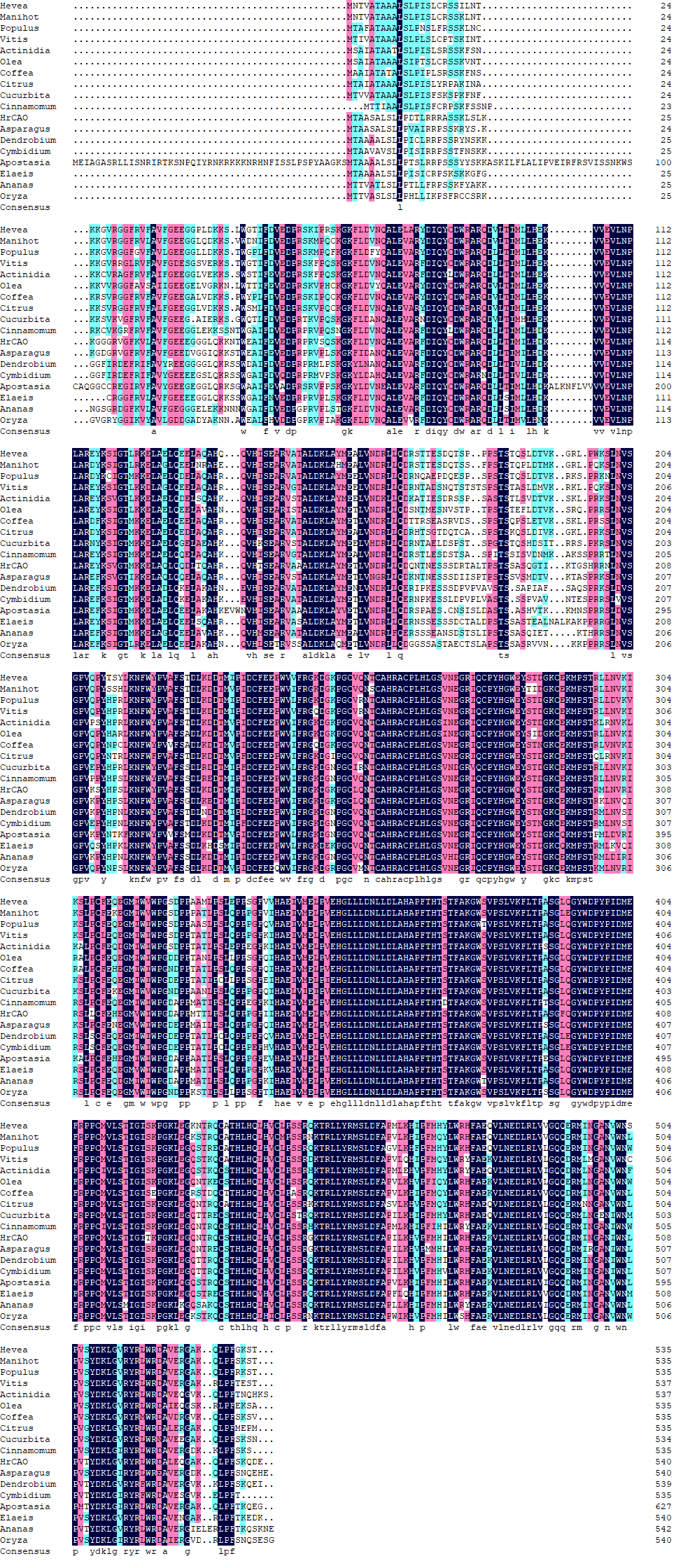

Supplement: Supplementary file 3 — Additional file 3: Fig. S1. Alignment and evolutionary relationship results of HrHEMA from different species. [file 12870_2020_2805_MOESM3_ESM.tif]

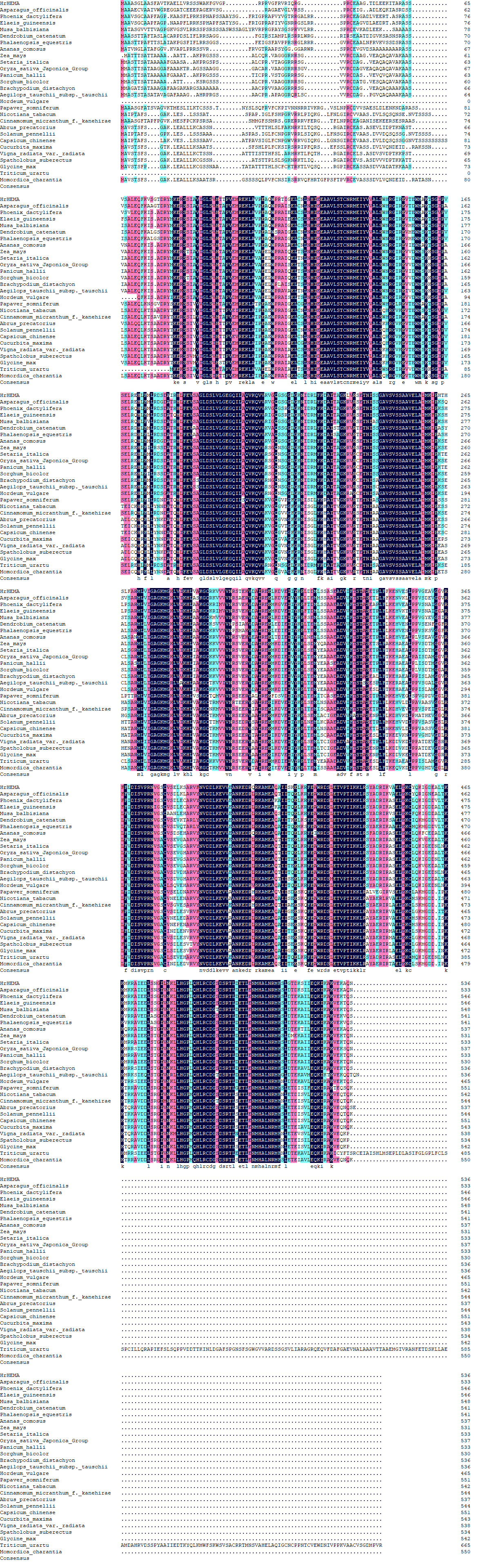

Supplement: Supplementary file 4 — Additional file 4: Fig. S2. Alignment and evolutionary relationship finding of HrPOR from different species. [file 12870_2020_2805_MOESM4_ESM.tif]

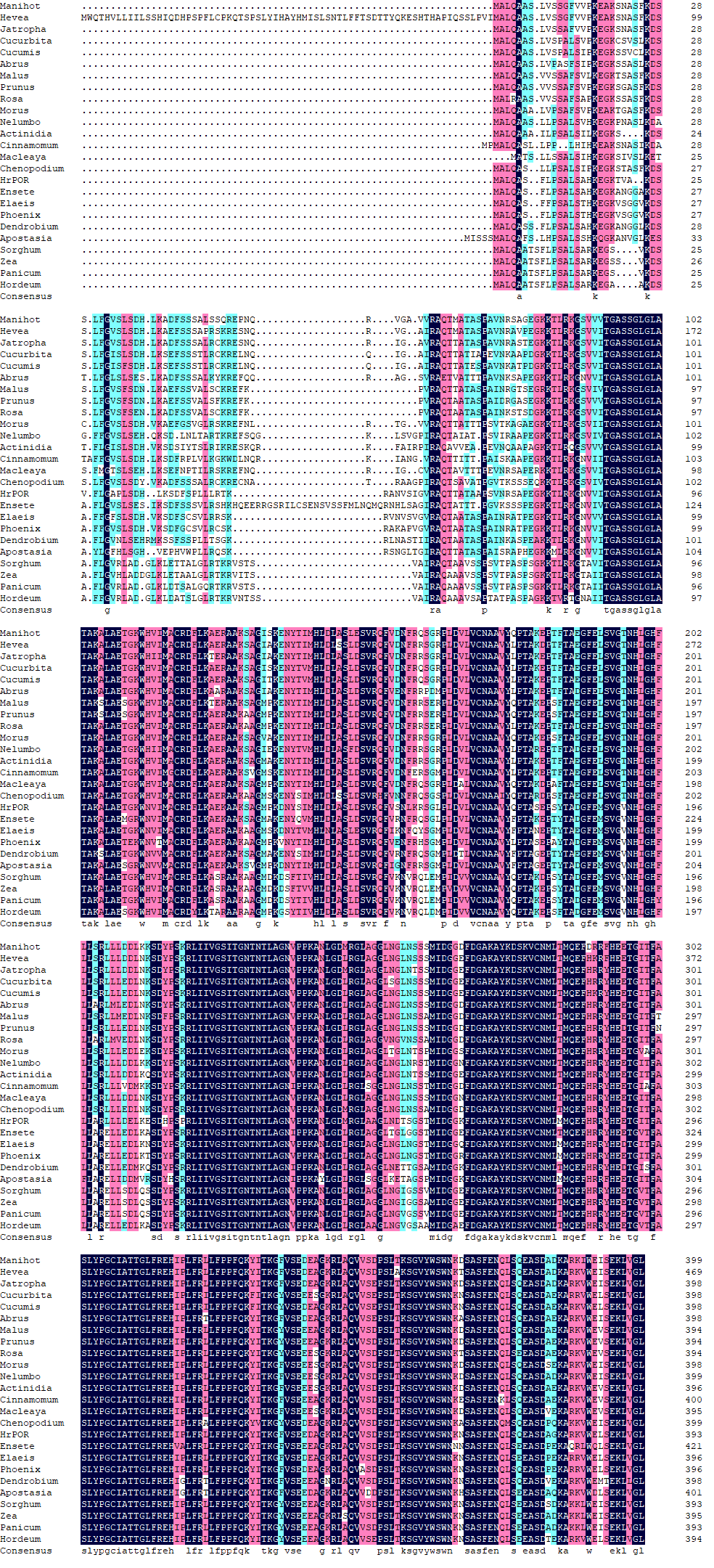

Supplement: Supplementary file 5 — Additional file 5: Fig. S3. Alignment and evolutionary relationship results of HrCAO from different species. [file 12870_2020_2805_MOESM5_ESM.tif]
